# The orientation selectivity of face identification

**DOI:** 10.1038/srep34204

**Published:** 2016-09-28

**Authors:** Valerie Goffaux, John A. Greenwood

**Affiliations:** 1 Research Institute for Psychological Science, Université Catholique de Louvain, Belgium; 2 Institute of Neuroscience, Université Catholique de Louvain, Belgium; 3 Department of Cognitive Neuroscience, Maastricht University, The Netherlands; 4 Experimental Psychology, University College London, United Kingdom

## Abstract

Recent work demonstrates that human face identification is most efficient when based on horizontal, rather than vertical, image structure. Because it is unclear how this specialization for upright (compared to inverted) face processing emerges in the visual system, the present study aimed to systematically characterize the orientation sensitivity profile for face identification. With upright faces, identification performance in a delayed match-to-sample task was highest for horizontally filtered images and declined sharply with oblique and vertically filtered images. Performance was well described by a Gaussian function with a bandwidth around 25°. Face inversion reshaped this sensitivity profile dramatically, with a downward shift of the entire tuning curve as well as a reduction in the amplitude of the horizontal peak and a doubling in bandwidth. The use of naturalistic outer contours (vs. a common outline mask) was also found to reshape this sensitivity profile by increasing sensitivity to oblique information in the near-horizontal range. Altogether, although face identification is sharply tuned to horizontal angles, both inversion and outline masking can profoundly reshape this orientation sensitivity profile. This combination of image- and observer-driven effects provides an insight into the functional relationship between orientation-selective processes within primary and high-level stages of the human brain.

The remarkable efficiency of human face recognition presents a challenge to our understanding of the visual system. Research on the face inversion effect (FIE) indicates that this efficiency is likely derived from observer-driven biases at high-level stages of visual processing that are selective for the higher-order properties of *upright* faces (e.g., features like eyes, nose, and mouth and their canonical spatial arrangement within the face^1^,^2^,^3^,^4^,^5^). How such high-level specialization emerges from earlier stages of visual encoding is largely unknown.

In the brain, visual processing begins with the encoding of local edge orientation in primary visual cortex (V1). The responses of V1 neurons typically reveal a preferred orientation that produces a peak level of response, with decreasing responsiveness for edges angled away from the preferred orientation^6^,^7^. The function relating V1 neuronal responses to orientation is a Gaussian whose bandwidth reflects the sharpness of the tuning to orientation. Electrophysiological recordings in monkey V1 have shown that the tuning bandwidth for most single neurons is between 20 and 50° full-width at half-maximum^8^,^9^. Mechanisms with similar tuning bandwidths have been argued to underlie the human perception of tilt contrast and the tilt aftereffect^10^,^11^. However, it is clear that our efficiency at processing faces does not arise at this computational level. The inversion of a face in the picture plane produces a dramatic impairment in face perception^12^ despite the fact that it preserves the orientation content of the image (aside from flipping its sign) and should not therefore significantly alter V1 responses at the population level^13^.

Recent evidence nonetheless suggests that the high-level mechanisms selectively recruited for upright face identification are orientation-selective. When upright faces are filtered to preserve information in narrow orientation ranges (Fig. 1a), human identification performance peaks in the horizontal band, declines progressively as stimulus orientation departs from horizontal, and reaches its minimum in the vertical range^14^. With face inversion, however, human sensitivity to facial identity becomes comparable for horizontal and vertical image structure. These findings suggest that high-level face-specialized mechanisms are particularly sensitive to the horizontal range of facial information^15^,^16^,^17^. Accordingly, the removal of the horizontal components of the facial image has been found to disrupt face-specialized processing (as marked by the FIE) in several of the high-level visual regions responding preferentially to faces in the human and monkey brain (i.e., FFA^13^,^18^,^19^).

Our recent developmental studies indicate that the maturation of face-specialized processing over the lifespan (indexed by an increase in FIE size) depends on the extensive experience humans acquire at encoding the horizontal information conveyed by upright faces^20^ (see also refs 21 and 22). The horizontal advantage is likely to arise because this orientation range gives access to the most stable and informative facial cues to identity, namely the vertical arrangement of the horizontally-oriented bands containing the eyes, brows, and mouth^2^,^23^,^24^,^25^,^26^.

These findings suggest that the high efficiency of face recognition is driven by a heightened sensitivity to the horizontal orientation range of the facial image. However we are still missing a complete picture of the orientation sensitivity profile of face-specialized processing. Most previous studies have examined only a limited range of orthogonal orientations (e.g., horizontal versus vertical orientation bands, e.g., refs 17,21 and 27) therefore preventing the systematic quantification of the tuning function potentially relating face processing to orientation. Only two studies have manipulated the orientation content of face stimuli in a semi-continuous manner. Dakin and Watt^14^ found a Gaussian tuning profile for the recognition of upright orientation-filtered face stimuli. However, without the concurrent measurement of the selectivity for inverted face recognition it is difficult to unequivocally attribute the reported results to the specialized mechanisms that are selectively recruited for the processing of upright faces. In the second study^16^, contrast thresholds in a face identification task revealed a peak in the horizontal range, but the overall pattern did not show the same Gaussian modulation as a function of orientation. Although this was likely due to the particularly challenging conditions required by the authors’ ideal observer approach, it nonetheless casts doubt on the precise shape of the orientation sensitivity for face-specialized processing.

The aim of the present study was therefore to systematically characterize the orientation sensitivity profile of upright and inverted face processing and to yield novel quantitative insights into how the specialization of upright face processing emerges in the visual system. Theoretically, if we assume the underlying sensitivity profile to be Gaussian in shape, the specialization of upright compared to inverted face processing (as indexed by the FIE) may arise through changes in four different tuning properties (Fig. 1b): peak location (i.e., the orientation range with highest sensitivity), peak amplitude, bandwidth, or base amplitude (i.e., an overall change in sensitivity at all orientations). Past evidence enables us to preclude some of these potential accounts immediately. For example, a peak shift is unlikely on its own, as it would not lead to the overall decrease in human sensitivity that conventionally results from inversion. Further, considering previous reports of a differential FIE size in the horizontal and vertical ranges of face information, we do not expect inversion to result in a pure broadening or downward shift of the tuning curve. The specialization of face processing may therefore result from a combination of these tuning specificities.

An additional source of uncertainty about the orientation selectivity of face-specialized processing relates to the influence of external facial features such as facial outline. It is standard practice to mask the outline of face stimuli in order to investigate the contribution of internal facial features to face perception. Accordingly, past studies on the orientation selectivity of face perception have employed outline-masked face stimuli, with the exception of Dakin and Watt^14^ who conserved all external facial cues (hair, ears, neck, outline, paraphernalia, and even scene background). As a matter of fact, facial outline contributes to face identification^28^,^29^,^30^ and its processing recruits face-specialized mechanisms^28^,^31^,^32^,^33^. Since outline cues are mainly conveyed by non-horizontal orientation ranges, their masking attenuates image amplitude at oblique/vertical orientations. This is shown for our stimuli in Fig. 2, using methods similar to prior studies^34^ (see methods for details). It may be that the attenuation of this oriented structure has led to an overestimation of the importance of horizontal information for face identification. It is therefore important to take facial outline into account in order to advance knowledge about the orientation tuning properties of face-specialized processing.

We therefore investigated the influence of inversion and outline masking on face processing in the orientation domain. Face images with masked or naturalistic outlines were filtered using 15°-wide Gaussian filters centered on orientations from vertical to horizontal in steps of 22.5° (Fig. 1a). Participants discriminated face image pairs at both upright and inverted planar orientations in the different filtering conditions. We then estimated the parameters of the tuning function that relates face-matching performance to image orientation content.

## Results

### The influence of inversion and outline masking on sensitivity in the horizontal and vertical ranges

For the sake of comparison with our previous work, where the FIE was tested in the horizontal and vertical orientation ranges only, we first ran an ANOVA with picture-plane and filter orientation as within-subject factors and outline masking as a between-subject factor. In agreement with previous findings, we found significant main effects of filter orientation and planar orientation (F(1,73) = 166, p < 0.0001, η^2^ = 0.695 and F(1,73) = 149, p < 0.0001, η^2^ = 0.671). These effects reflect the finding that our participants were most sensitive to identity differences in the horizontal (vs. vertical) orientation range and in upright (vs. inverted) viewing conditions (Fig. 3a). The interaction between these two factors was also significant (F(1,73) = 9.95, p < 0.002, η^2^ = 0.12).

We explored the significant picture-plane orientation by filter orientation interaction using Bonferroni-corrected paired t tests and found that the inversion effect was significant in both horizontal and vertical orientation ranges (t(1,74) = 11.91, p < 0.0001 and t(1,74) = 6.66, p < 0.0001, respectively). Nonetheless, the difference in sensitivity between upright and inverted conditions was larger in the horizontal than the vertical range (t(1,74) = 3.18, p < 0.002). We also found significantly better sensitivity in the horizontal than the vertical range both in upright and inverted viewing conditions (t(1,74) = 12.15, p < 0.0001 and t(1,74) = 7.31, p < 0.0001, respectively). The horizontal-vertical sensitivity difference was largest when faces were viewed upright (t(1,74) = 3.18, p < 0.002). In contrast, outline masking had no significant influence on sensitivity in the horizontal and vertical orientation ranges (F < 1, p = 0.4, η^2^ = 0.01), nor did it modulate the influence of picture-plane and filter orientation upon matching sensitivity (Fs < 1.9, ps > 0.18, η^2^ < 0.025).

**Figure 1:**
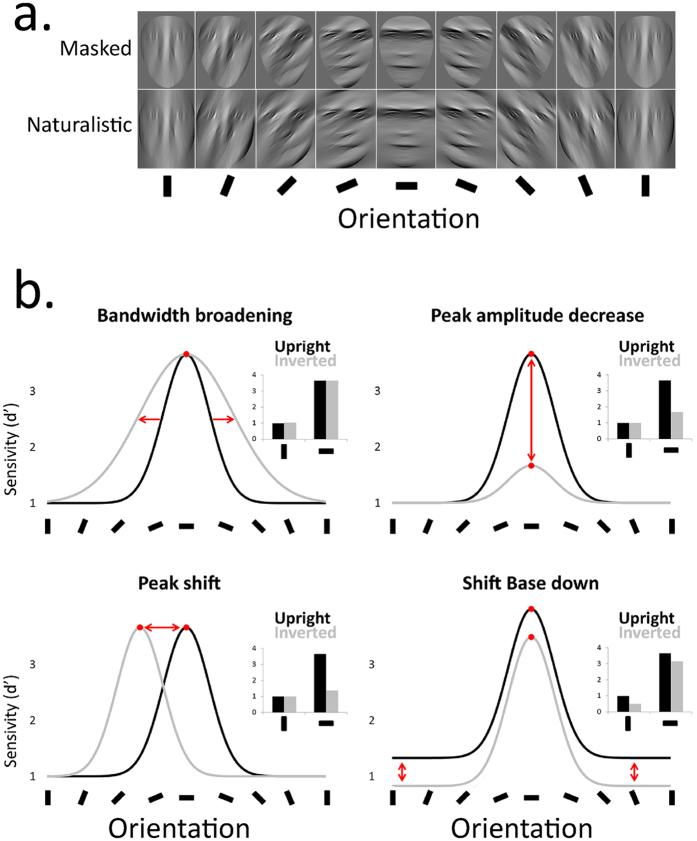
(**a**) Example stimuli used in the present experiments. Greyscale face pictures were filtered in steps of 22.5° from vertical (here, 0°) to horizontal orientations (90°) and back to vertical again. The outlines of filtered faces were either seen through a rounded triangular aperture (i.e., ‘masked’), or not (‘naturalistic’). (**b**) Line graphs illustrate the Gaussian tuning function describing the hypothetical pattern of human sensitivity (d’) to face identity as a function of orientation range, both for upright faces (black line) and for faces inverted in the picture plane (grey line). Bar graph insets represent the same hypothetical data with an exclusive focus on cardinal orientations.

Altogether, the above results replicate previous findings to show that the horizontal range of face information carries more useful cues for face identification than the vertical range, and that the extraction of horizontal identity cues is particularly vulnerable to inversion. We therefore confirm that the specialization of face identification pertains to its stronger reliance on horizontal than vertical face cues. We found no significant influence of outline masking on the processing of horizontal and vertical face information. However, because they are restricted to a few discrete orientation ranges, the above analyses and results may not be sufficiently sensitive to reveal the influence of outline masking on the orientation selectivity of face processing. They also fall short of explaining exactly how the latter is modulated by picture-plane inversion (Fig. 1b). In the next analyses, we investigate how inversion and outline masking affects the function relating face identification performance to orientation.

### The influence of inversion and outline masking on orientation sensitivity

The variation in matching sensitivity for upright faces as a function of filter orientation was described by a Gaussian function, with best-fitting parameters that gave a peak at around 90° and a full-width at half-maximum of 30° and 23° for naturalistic-outline and masked-outline faces, respectively (Fig. 3b; Table 1). Sensitivity variations for inverted face recognition were similarly well described by Gaussian functions, with peak values again around 90° and FWHM values of 55.7° and 47.4° for naturalistic- and masked-outline faces, respectively.

To explore the influence of inversion (i.e., picture-plane orientation) on the orientation-tuning curve, we bootstrapped the difference between upright and inverted Gaussian parameter estimates, separately for naturalistic- and masked-outline faces (Table 1). These analyses revealed that in both naturalistic- and masked-outline conditions inversion significantly shifted the base of the tuning curve downwards by approximately 50%, reduced peak amplitude by about 20% and doubled tuning bandwidth. The FIE in each parameter estimates was comparable across masked- and naturalistic-outline conditions (rightmost columns of Table 1).

Next, we investigated the influence of outline masking on the orientation tuning of upright and inverted face identification performance (see Table 1). For both upright and inverted faces, the masking of facial outline slightly but significantly displaced peak location (by 2°) and decreased tuning bandwidth (by 22% in upright and by 14% in inverted condition). For upright faces, it additionally impaired sensitivity across all orientation ranges by 9%. However, when compared directly, the influence of outline masking on parameter estimates was comparable between upright and inverted conditions.

In sum, we found that variation in the sensitivity to facial identity as a function of orientation structure is well described by a Gaussian function, sharply tuned to the horizontal range. Inversion impaired sensitivity across all orientations but more so in the horizontal range (i.e., peak amplitude was decreased). Inversion also drastically disrupted the orientation selectivity of face processing (i.e., broadening the tuning bandwidth) so that a greater range of orientation values contributed more similarly to face processing in inverted than upright viewing conditions. In contrast to previous analyses, which did not reveal any influence of outline masking on performance, the present analyses showed that the masking of facial outline increased the selectivity of face processing to the horizontal band (i.e., via a decreased tuning bandwidth). In the next section, we take a finer-grained look at the orientation tuning of face-specialized mechanisms via the analysis of the function relating the FIE to filter orientation.

### FIE orientation tuning

Group-level FIE size was estimated in each planar-orientation and outline masking condition based on 10,000-bootstrapped Cohen’s d coefficients. As shown on Fig. 4, FIE size followed a difference of Gaussian (DoG) function with a peak value close to horizontal for both naturalistic- and masked-outline faces (Fig. 4 and Table 2). When moving from horizontal towards vertical extremes, FIE size dropped abruptly to reach two minima in the right- and left-oblique ranges. After reaching its left and right oblique minima, FIE size increased slightly when reaching the vertical range.

Outline masking increased the amplitude of the peak of the DoG FIE tuning curve by 21% and shifted the whole DoG FIE function downwards by 15%. In addition, it decreased the bandwidth of the DoG FIE tuning curve by 9%, which resulted in closer oblique minima in the masked than the naturalistic condition. Outline masking further boosted the amplitude of the right-oblique minimum by 30% (i.e., 1^st^ minimum on Fig. 4). In contrast, outline masking negligibly but significantly reduced the amplitude of the left-oblique minimum (i.e., 2^nd^ minimum). This suggests that right-oblique more importantly than left-oblique orientations contribute to the specialized processing of (upright) facial outline, at least for the faces in our stimulus set.

## Discussion

Our findings provide further evidence that the human visual system deciphers facial identity most efficiently based on horizontally oriented information. The fact that a high-level perceptual ability like face recognition could be orientation-selective, as in earlier stages of visual processing, opens interesting avenues to study the way that visual signals are transformed from primary to high-level stages of the visual system^13^,^35^. However, a systematic quantitative characterization of the orientation sensitivity profile for face processing was still missing – previous studies have mainly relied on the comparison of performance between selected orientation values (Fig. 3a). The present work has filled this gap by investigating facial identity discrimination over a more complete range of orientation values. We compared the orientation sensitivity profile for the recognition of both upright and inverted faces in order to isolate genuine, (upright) face-specialized processes from more low-level contributions. We further examined the way that the masking of facial outline, a method conventionally used to restrict processing to inner facial cues, impacts the orientation tuning profile of face processing and revealed clear observer-driven modulations in sensitivity as a result.

**Figure 2:**
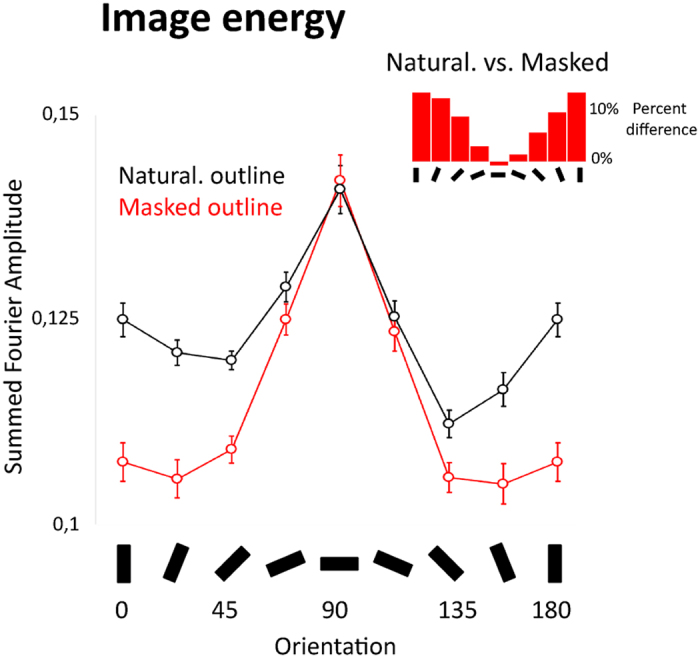
Fourier energy analyses for our stimuli. Outline masking alters the face image at non-horizontal orientation ranges. The line graph shows the summed Fourier amplitude values in distinct orientation ranges, separately for naturalistic and masked outline conditions. Error bars are the 95% confidence intervals (see Methods for image analysis details). The inset bar graph shows the difference in energy obtained by subtracting energy in the masked outline condition from the naturalistic condition in each orientation range.

We report that human sensitivity to identity is strongly tuned to horizontal angles (consistent with previous findings^14^), with a bandwidth of about 25° FWHM (see Table 1). This tuning bandwidth is remarkably comparable in size to previous reports on the average orientation selectivity in V1 neurons, as well as the breadth of psychophysical tilt after-effects^10^,^11^,^36^,^37^. It may be that the orientation selectivity of V1 sets a lower limit on the range of orientations that can be enhanced to more efficiently recognise faces. That is, at the image level, faces clearly have the most energy within the horizontal bands (Fig. 2). For the visual system to enhance sensitivity to this information within face-selective stages, the lower limit on the range of orientations that can be selectively enhanced may be the values set by V1 orientation selectivity.

Prior studies have found that inversion affects facial identity processing with horizontally-filtered faces to a far greater extent than with vertically-filtered faces^16^,^17^,^19^. This was taken to suggest that face processing specialization resides in a boost of sensitivity to the horizontal facial information, though the exact tuning characteristics underlying such specialization were still undetermined (Fig. 1). Here we show that the orientation sensitivity profiles of upright and inverted face processing diverge at three levels: the base, peak amplitude, and bandwidth. Hence, inversion decreased identity discriminability across all orientations (i.e., a downward base shift) but to a significantly greater extent in the horizontal range (i.e., reduced peak amplitude). Inversion additionally doubled the bandwidth of the tuning curve, literally reshaping the orientation sensitivity profile of face identification. These results indicate that what makes (upright) face identity processing special is not only its boosted sensitivity to horizontal orientations but also its *sharper* tuning to the horizontal range.

In line with the above findings, the difference between upright and inverted sensitivity, expressed as the magnitude of the Face Inversion Effect (FIE), was found to be finely tuned to horizontal angles (with a bandwidth of about 20°). This pattern was well-described by a Difference-of-Gaussian function, which reached minima at the right and left oblique angles (Fig. 4), with a rise in magnitude for the vertical ranges. In other words, the orientation range with the least change in sensitivity with inversion is not vertical but rather the adjacent oblique orientations. A simple comparison between vertical and horizontal orientation ranges does not therefore convey the full extent of the FIE. Rather, it seems that it is the orientation sensitivity profile as a whole that underlies our face processing specialization.

Although inversion decreased sensitivity to horizontal angles and increased the breadth of the sensitivity profile, it did not shift the peak in orientation tuning away from horizontal. This preservation of the horizontal advantage for inverted faces hints at the existence of a general horizontal bias for visual recognition. In a related vein, Goffaux and Dakin^17^ reported that, as with faces, the discrimination of car pictures is better at horizontal than vertical angles. This general horizontal advantage may be explained by the fact that the structure of stimuli like (upright and inverted) faces and cars is predominantly biased towards horizontal angles, as we show for our stimuli in Fig. 4, and as shown by others^34^ (though differences have been reported for other stimulus types^38^). Another aspect likely conferring a special status to horizontal information for visual recognition in general is that the left-right bilateral symmetry of stimuli is particularly salient in the horizontal structure of the image^39^,^40^,^41^. However, the salience of this bilateral symmetry applies to both upright and inverted face images equally, meaning that these image properties cannot fully account for the peculiar orientation sensitivity profile when processing upright faces.

Rather, the modulation of the base, peak amplitude, and bandwidth of facial orientation selectivity by inversion must stem from observer-driven mechanisms specialized for the processing of the higher-order properties of upright faces. That is, the detection of the main facial features (e.g., eyes, eyebrows, and mouth) in a canonical upright arrangement would activate a sharp tuning to the horizontal range^14^,^16^,^17^,^42^.

The horizontal bias in face processing has so far been demonstrated based on facial stimuli viewed through circular or elliptical apertures (with one exception^14^). However, masking the natural facial outline in this way has been shown to deteriorate the efficiency of face processing^28^,^29^,^30^. Accordingly, we found that outline masking made upright face identification less efficient overall (seen in the downward shift of the FIE tuning curve in Fig. 4). Furthermore, since the facial outline is mainly represented in the oblique and vertical angles, its masking reduces energy solely in these ranges (Fig. 2). We were therefore concerned that this manipulation would artificially exaggerate the contribution of horizontal angles to face processing. Our results suggest that this is true to some extent, as outline masking made the processing of both upright and inverted faces more sharply tuned to the horizontal range. We addressed the influence of outline masking on face-specialized processing more specifically by analyzing the orientation sensitivity profile of FIE size for naturalistic- and masked-outline conditions separately (Fig. 4). FIE size peaked in the horizontal range irrespective of outline masking, further corroborating the generalizability of this tuning property. Outline masking diminished FIE size across all orientations, in line with past evidence that contour cues contribute to face-specialized processing^32^. It additionally increased the (horizontal) peak amplitude and sharpened the bandwidth of the FIE orientation sensitivity profile. In other words, masking facial outline inflated the horizontal selectivity of the FIE both by increasing the dependence to the horizontal range and diminishing the contribution of other orientations. FIE size was most drastically reduced by outline masking at right-oblique angles, resulting in an asymmetric orientation sensitivity profile when viewing outline-masked faces. In contrast, when naturalistic contour variations were preserved in the stimulus, the orientation tuning was symmetric with more comparable FIE size in the left- and right-oblique angles (Fig. 4 and Table 2). This suggests that the breadth of orientation selectivity for faces becomes broader as more information (e.g., outline) is added to the image. Contour masking should therefore be used with caution as it distorts the properties of the face image, impairs face processing efficiency and disengages face-specialized processing mainly due to the disrupted processing of the oblique elements of the outline. Precisely where in the image these tilted contour cues derive (i.e., lower-right chin, and/or upper-left hairline) and how these cues respectively affect the perception of facial configuration are unanswered questions.

It is interesting to note that while the masking of naturalistic outlines produced a strong decrease in energy in the vertical orientation range, as well as the adjacent oblique angles (Fig. 2), the primary difference in FIE magnitude produced by these manipulations was for the right oblique angles, with little-to-no effect in the vertical range (Fig. 4). In other words, adding information in orientation ranges that are not prioritised by face-selective identification processes (i.e. around vertical) had no effect on FIE orientation sensitivity profile (Fig. 4). Through the combination of orientation filtering and variations in image content we can therefore visualize the observer-driven biases of face recognition in action.

Since we used only one picture per face identity, participants did not have to match identity across different poses as in everyday life. One may therefore argue that face identification mechanisms were not fully engaged here and that participants relied on picture-matching strategies. But the large inversion effect found around the horizontal range indicates that this account is unlikely as inversion should have little-to-no effect on picture matching. Moreover, we have previously shown that the ability to generalize face identity across viewpoints (e.g., between frontal and ¾ view) preferentially relies on horizontal information^17^. We explained this by the fact that when a head rotates in the horizontal plane the vertical arrangement of the main features (e.g., the eyes-mouth distance) as well as the horizontal structure of those features is preserved better than facial cues at other orientations (e.g., nose, eye separation^2^). Therefore we predict that presenting face identities under varying viewpoints would render face identification performance even more sharply tuned to the horizontal range due to the particular stability and diagnosticity of the identity cues conveyed in this range.

**Figure 3:**
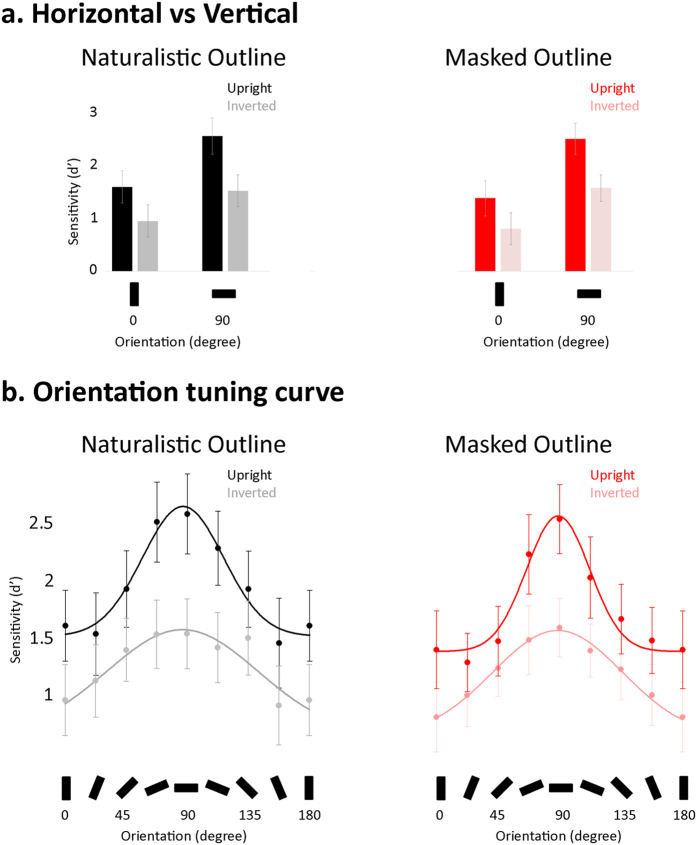
(**a**) Performance with horizontally- versus vertically-filtered face images. Group-averaged face matching sensitivity is plotted in the horizontal and vertical orientation ranges in each outline by picture-plane orientation condition. Error bars are 95% confidence intervals. (**b**) The orientation sensitivity profile of identification performance. Group-averaged face matching sensitivity is plotted as a function of filter orientation separately for each outline by picture-plane orientation condition. Dots and lines illustrate group averages and the best fitting Gaussian tuning functions, respectively. Error bars are 95% confidence intervals.

Altogether, our quantitative approach has revealed (1) that face identification is sharply tuned to horizontal angles, and (2) that inversion and outline masking profoundly reshape this orientation sensitivity profile. Compared to past studies that focused on selected orientation values (as in Fig. 3a), we present richer information on the complex relationship that exists between the sampling of primary visual information and high-level and specialized processing in the human brain. The similarities in orientation sensitivity profile between basic visual functions (e.g., tilt perception) and high-level face identification indicate there might be more continuity in the features encoded at primary and high stages of visual processing than is commonly assumed, opening new leads for a more comprehensive approach to human vision. That is, face perception research often relies on the comparison between a few divergent stimulus conditions (aligned versus misaligned top and bottom face halves^33^, features isolated from versus embedded in a congruent versus incongruent face context^43^,^44^,^45^, etc). Although such methods have yielded important insights, the present work underlines the potential for more quantitative approaches to reveal functional aspects of face processing specialization^46^,^47^.

## Methods

### Subjects

We tested 75 healthy young adults (15 males; 2 left-handers; age range: 21 ± 1.7 years). All subjects reported normal or corrected-to-normal visual acuity, provided informed consent prior to participation, and were naïve to the purpose of the experiments. Experimental protocols adhered to the Declaration of Helsinki and were approved by the local ethics committee (Faculty of Psychology and Neuroscience, Maastricht University; ECP-7-11-2004-A1).

### Stimuli

We used forty unfamiliar greyscale pictures of faces (190 by 250 pixels; half male) posing in a neutral expression (Fig. 1a). Face pictures were cropped in Adobe Photoshop to remove hair, neck and ears. Models were alumni Psychology students (aged 18–25 years) of the Université Catholique de Louvain (Belgium). First we normalized face images to obtain a root-mean square (RMS) contrast of 1. Next, images were fast Fourier transformed, with the amplitude spectra multiplied with wrapped Gaussian filters (with a standard deviation of 15°) centered on 0° to 157.5° (cf.^14^,^17^; see Fig. 1a for example), where 0° is vertical and 90° horizontal. After the inverse Fourier transform, the luminance and RMS contrast of all resulting images was adjusted to match the average luminance and RMS contrast of the original image set. We created outline-masked stimuli by embedding the output faces in a rounded triangular aperture to eliminate and mask the outer facial outlines (Fig. 1a). Aperture shape was identical across outline-masked stimuli. In contrast, the outer shape of the naturalistic-outline stimuli was created through the removal of hair, neck and ears, and varied across exemplars. Inverted stimuli were created by vertically flipping each image. During the experiment, images were framed by a 3 pixel-wide light grey square border (at 75% of the maximum luminance).

All testing was performed on an LCD monitor with 1024 × 768 pixels resolution. At a viewing distance of 60 cm, stimuli were presented on a mid-grey background and subtended a visual angle of 6.6 by 8.7°.

### Image analyses

To consider the effect of outline masking at the image level, we computed the Fourier energy of our filtered stimuli in each outline condition. Images were first padded with grey values to extend image dimensions to 512 × 512 pixels. A two-dimensional fast Fourier Transform was then taken to obtain the amplitude and phase spectra for each image. Similar to the filtering performed on our stimuli, amplitude spectra were multiplied with wrapped Gaussian filters, with peak orientations between 0–157.5° in 22.5° steps and a standard deviation of 15°. Amplitude values at all spatial frequencies within each of these orientation ranges were summed and divided by the overall amplitude sum for each image. In order to reduce pixel- and image-based artifacts, we conducted the analyses with each image rotated by 0–180° in 22.5° steps, cropping the result to preserve the image size^48^. Filter orientations were similarly rotated, with the final outputs then rotated back to be consistent with an upright face. The final amplitude sum for each orientation range in each image was then taken as the average of all the rotated image analyses. The output of these image analyses is shown in Fig. 2.

These amplitude values were submitted to a repeated-measures ANOVA with outline masking and orientation range as within-subject factors (where each face is treated as a ‘subject’). The main effects of outline masking and orientation range and their interaction were all significant (Fs > 132, ps < 0.0001, η^2^s > 0.87). For both sets of faces, there was a clear peak in energy within the horizontal range. Naturalistically-outlined faces had higher energy around the vertical and the adjacent oblique ranges than masked faces, with no or little change around horizontal (Fig. 2).

### Procedure

In a delayed match-to-sample task, participants were instructed to decide whether two successive face identities matched (“same”) or not (“different”). Each trial started with the presentation of a white fixation cross at screen center for 300 ms, followed by the first face picture (i.e., the target face) for 500 ms. In “same” trials, the exact same image was presented twice. To prevent subjects from relying on local image comparison strategies, the screen position of the target face was randomly jittered around the screen center by 20 pixels in both x and y planes on each trial. A rectangular mask of the same size as face images (190 by 250 pixels) filled with Gaussian white noise immediately followed for 200 ms, with the same luminance, contrast, and screen location as the target. The second face (i.e., probe face) then appeared for 400 ms at screen center. The trial ended by the presentation of a white fixation cross at screen center (average duration of 2000 ms, range: 1750–2350 ms).

Participants indicated whether target and probe faces were the “same” or “different” by pressing the “s” and “l” keys with the left- or right-hand index finger. Responses given later than 3000 ms after the onset of the probe face were coded as errors.

Before the test began, subjects were randomly assigned to group A or B. In group A, naturalistic variations of facial outline across the different identities were preserved, whereas facial outline was masked in group B. Participants first practiced the task with 70 trials randomly selected from the list of the experimental trials. During practice, resting pauses were provided every 10 trials along with written feedback on their performance accuracy. Practice was repeated until subjects achieved at least 70% accuracy. During the experiment, pauses with the accuracy feedback were provided every 32 trials. Pause duration was self-paced.

There were 4 experimental factors (Filter orientation: 0, 22.5, 45, 67.5, 90, 112.5, 135, or 157.5°; Picture-plane orientation: upright, inverted; Outline masking: present, absent; Similarity: same, different) and a total of 32 experimental conditions. Since there were 16 trials per conditions, the experiment was 512 trials in total (approximate duration: 42 minutes). Picture-plane orientation (upright, inverted) varied randomly every 8 trials whereas all other factors randomly varied at the trial level.

### Data analyses

We recorded the accuracy and the reaction time of participants’ manual responses. However, because task performance was unspeeded (with subjects instructed to emphasise accuracy over speed), we focus here on the accuracy data. To this end, we estimated individual sensitivity (d’) based on hit and correct rejection rates in each condition, following the log-linear procedure^49^.

We first focused our analyses on the horizontal and vertical filter orientation ranges. We submitted sensitivity measures to a three-way mixed effects ANOVA with outline masking as a between-subject factor, and with filter orientation and planar orientation as within-subject factors. For post-hoc two-by-two comparisons, we used paired t tests. Statistical threshold was Bonferroni-corrected for the total number of comparisons, resulting in an alpha value of 0.0083.

Next, we investigated the effects of picture-plane orientation and outline masking more continuously as a function of filter orientation (from 0° to 180° in steps of 22.5°). The group averaged sensitivity profile was computed separately for upright and inverted picture-plane orientation conditions, and likewise for naturalistic and masked outline conditions. Performance in all cases was well described by Gaussian functions fit to the data with four free parameters (Fig. 3b). Best-fitting parameters for peak location, peak amplitude, bandwidth and overall height at the group level were estimated via bootstrapping (n = 10,000 repetitions; Fig. 3b). To exclude outliers, analyses were carried on the 25%-trimmed bootstrapped estimates (two-tailed^50^). This procedure was run separately in each Picture-plane orientation by Outline masking condition (Table 1). To explore the influence of inversion on the orientation tuning curve separately derived for naturalistic and masked outline conditions, we subtracted inverted from upright trimmed parameter estimates (Table 1) separately for naturalistic and masked outline conditions. Upright vs inverted differences were reported as being significant if the 95% confidence interval around the mean for each condition excluded a value of zero. We used the same procedure to explore the influence of outline masking on the orientation tuning curve (Masked vs Naturalistic) for upright and inverted conditions separately (Table 1).

**Figure 4:**
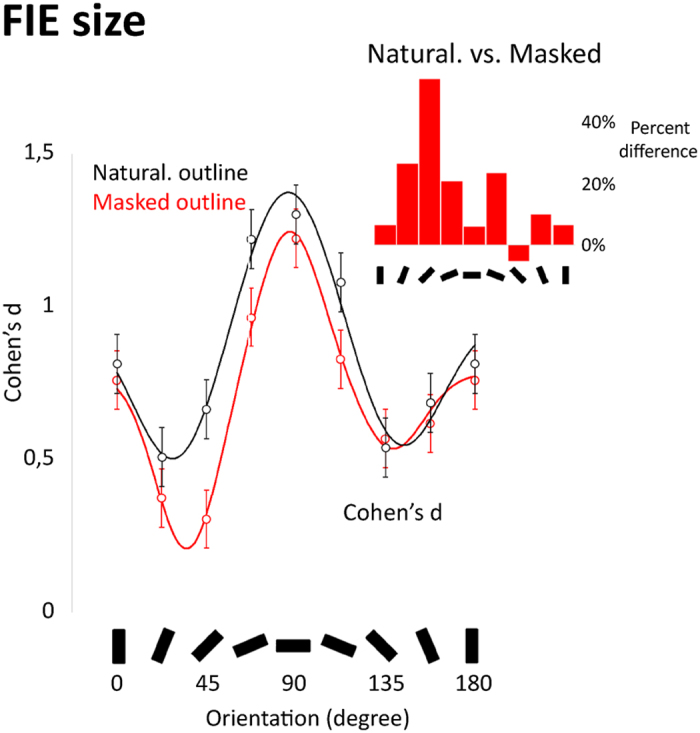
Group-averaged Face Inversion Effect (FIE) size in Cohen’s d as a function of orientation for masked and naturalistic contour conditions. Dots and lines illustrate group averages and the best fitting difference-of-Gaussians (DoG) functions, respectively. Error bars are 95% confidence intervals. The inset shows the difference in FIE size obtained by subtracting FIE size in the masked outline condition from the naturalistic condition in each orientation range.

Group-level FIE size was estimated in each orientation and outline masking conditions based on 10,000-bootstrapped Cohen’s d coefficients. Cohen’s d coefficients quantified not only the strength but also the consistency of the FIE at the sample level. They were computed by subtracting the averaged inverted from upright sensitivity and dividing the difference by the pooled standard deviation for each bootstrapped sample (Fig. 4). The function relating FIE size (in Cohen’s d) to orientation resembled a difference of Gaussians (DoG) function, i.e. a central Gaussian function bilaterally flanked by two minima. The function was fit with 7 free parameters: the location, amplitude, and bandwidth of the central peak, the difference in location and amplitude of the first and second minima, and the overall baseline height. Fits estimated via bootstrapping (n = 10,000 repetitions). After trimming the upper and lower 25% of the DoG parameter estimates, we computed the difference between Masked and Naturalistic outline masking conditions. A difference was reported as being significant if its respective 95% CI excluded a value of 0 (Table 2).

## Additional Information

**How to cite this article**: Goffaux, V. and Greenwood, J. A. The orientation selectivity of face identification. *Sci. Rep.* **6**, 34204; doi: 10.1038/srep34204 (2016).

## Figures and Tables

**Table 1:** Means and 95% CI of the Gaussian parameter estimates (n = 5,000 trimmed group-level bootstrapped estimates), for naturalistic and masked outlines in upright and inverted faces, separately.

Parameter		Naturalistic outline		Masked outline		Masked vs. Naturalistic	
		mean	CI	mean	CI	mean	CI
Peak location	Upright	86,7	[85,7 87,6]	88,8	[87,3 90,4]	1,95	[0,96 2,94]*
	Inverted	86,5	[84,5 88,4]	88,5	[86,1 91]	1,98	[0,14 3,82]*
	Upright vs. Inverted	0,19	[−2,33 2,71]	0,31	[−2,63 3,24]	0,12	[−3,8 4,03]
Peak amplitude	Upright	1,13	[1,05 1,2]	1,17	[1,11 1,24]	0,06	[0 0,11]
	Inverted	0,92	[0,8 1,04]	0,92	[0,68 1,16]	−0,04	[−0,15 0,08]
	Upright vs. Inverted	0,21	[0,04 0,37]*	0,25	[0,01 0,5]*	0,05	[−0,25 0,35]
Bandwidth	Upright	29,9	[28,4 31,5]	23,2	[21,4 24,9]	−6,69	[−7,92 −5,46]*
	Inverted	55,7	[51,1 58,1]	47,4	[34,3 60,5]	−12,43	[−18,01 −6,84]*
	Upright vs. Inverted	−25,81	[−30,08 −21,53]*	−24,25	[−37,52 −10,99]*	1,56	[−12,36 15,47]
Base	Upright	1,52	[1,46 1,59]	1,38	[1,32 1,45]	−0,13	[−0,18 −0,08]*
	Inverted	0,66	[0,52 0,79]	0,65	[0,38 0,92]	−0,05	[−0,26 0,16]
	Upright vs. Inverted	0,87	[0,69 1,04]*	0,74	[0,46 1,01]*	−0,13	[−0,46 0,2]

Mean and 95% CI of the Gaussian parameter difference between upright and inverted (for naturalistic and masked outlines, separately; n = 5,000 trimmed group-level bootstrapped estimates) and between naturalistic- and masked-outline (for upright and inverted faces, separately; n = 5,000 trimmed group-level bootstrapped estimates). Means and 95% CI of the difference in inversion effect across masked and unmasked conditions are also included. Asterisks denote significant differences at p < 0.05.

**Table 2:** Means and 95% CI of the parameter estimates of the Difference-of-Gaussians (DoG) function relating FIE size to orientation (n = 5,000 trimmed group-level bootstrapped estimates), for naturalistic and masked outlines, separately.

Parameter	Naturalistic outline		Masked outline		Masked vs. Naturalistic	
	mean	CI	mean	CI	mean	CI
Peak location	86,1	[85,8 86,5]	86	[85,4 86,7]	−0,08	[−0,9 0,7]
Peak bandwidth	19,2	[18,6 19,8]	17,3	[16,8 17,8]	−1,79	[−2,46 −1,12]*
Peak amplitude	0,44	[0,41 0,47]	0,55	[0,5 0,59]	0,12	[0,05 0,18]*
Base	0,97	[0,95 0,99]	0,81	[0,79 0,84]	−0,15	[−0,18 −0,12]*
Minima separation	112,4	[111,1 113,8]	99,9	[97,2 102,6]	−13,9	[−17,3 −10,4]*
1st minimum amplitude	0,53	[0,51 0,56]	0,75	[0,71 0,79]	0,22	[0,18 0,26]*
2nd minimum amplitude	0,48	[0,45 0,5]	0,35	[0,31 0,39]	−0,12	[−0,17 −0,08]*

Means and 95% CI of the masked vs. naturalistic difference of the trimmed DoG parameter estimates. Asterisks denote significant differences at p < 0.05.
