# Corrigendum: The orientation selectivity of face identification

**DOI:** 10.1038/srep46992

**Published:** 2018-06-05

**Authors:** Valerie Goffaux, John A. Greenwood

Scientific Reports 6: Article number: 3420410.1038/srep34204; published online: 09 28 2016; updated: 06 05 2018

In the original version of this Article, the authors erroneously reported the standard deviation (SD) of the Gaussian fits as the full width at half maximum (FWHM).

In the Abstract,

“Performance was well described by a Gaussian function with a bandwidth around 25°.”

now reads:

“Performance was well described by a Gaussian function with a standard deviation around 25°.”

In Table 1, the Parameter “Bandwidth” now reads “Standard Deviation”.

In the Results section, under the subheading ‘The influence of inversion and outline masking on orientation sensitivity’,

“The variation in matching sensitivity for upright faces as a function of filter orientation was described by a Gaussian function, with best-fitting parameters that gave a peak at around 90° and a full-width at half-maximum of 30° and 23° for naturalistic-outline and masked-outline faces, respectively (Fig. 3b; Table 1). Sensitivity variations for inverted face recognition were similarly well described by Gaussian functions, with peak values again around 90° and FWHM values of 55.7° and 47.4° for naturalistic- and masked-outline faces, respectively.”

now reads:

“The variation in matching sensitivity for upright faces as a function of filter orientation was described by a Gaussian function, with best-fitting parameters that gave a peak at around 90° and a standard deviation (SD) of 30° and 23° for naturalistic-outline and masked-outline faces, respectively (Fig. 3b; Table 1). Sensitivity variations for inverted face recognition were similarly well described by Gaussian functions, with peak values again around 90° and SD values of 55.7° and 47.4° for naturalistic- and masked-outline faces, respectively.”

In Table 2, the Parameter “Peak Bandwidth” now reads “SD”.

In the Discussion section,

“We report that human sensitivity to identity is strongly tuned to horizontal angles (consistent with previous findings^14^), with a bandwidth of about 25° FWHM (see Table 1). This tuning bandwidth is remarkably comparable in size to previous reports on the average orientation selectivity in V1 neurons, as well as the breadth of psychophysical tilt after-effects^10,11,36,37^.”

now reads:

“We report that human sensitivity to identity is strongly tuned to horizontal angles (consistent with previous findings^14^), with a standard deviation of about 25°, and a full-width at half-maximum of approximately 60° (see Table 1). This tuning bandwidth is within the upper range of previous reports of the orientation selectivity in V1 neurons, albeit broader than the typical bandwidth of psychophysical tilt after-effects^10,11,36,37^.”

In the Methods section, under the subheading ‘Data analyses’,

“Best-fitting parameters for peak location, peak amplitude, bandwidth and overall height at the group level were estimated via bootstrapping (n = 10,000 repetitions; Fig. 3b).”

now reads:

“Best-fitting parameters for peak location, peak amplitude, standard deviation (SD) and overall height at the group level were estimated via bootstrapping (n = 10,000 repetitions; Fig. 3b). Note that SD values can be converted to full-width at half maximum by multiplying the SD by ~2.355.”

In the same section,

“The function was fit with 7 free parameters: the location, amplitude, and bandwidth of the central peak, the difference in location and amplitude of the first and second minima, and the overall baseline height.”

now reads:

“The function was fit with 7 free parameters: the location, amplitude, and SD of the central peak, the difference in location and amplitude of the first and second minima, and the overall baseline height.”

These errors have now been corrected in the HTML and PDF versions of the Article.

